# P-1308. Assessing the Drug Resistance Index of E. coli and K. pneumoniae in Two Public Hospitals in uMgungundlovu District, KwaZulu-Natal

**DOI:** 10.1093/ofid/ofaf695.1496

**Published:** 2026-01-11

**Authors:** Sabiha Essack, Rumana S Moosa, Hafizah Y Chenia, Luther King Abia Akebe

**Affiliations:** Antimicrobial Research Unit, College of Health Sciences, University of KwaZulu-Natal, Durban, KwaZulu-Natal, South Africa; University of KwaZulu-Natal, Durban, KwaZulu-Natal, South Africa; University of KwaZulu-Natal, Durban, KwaZulu-Natal, South Africa; University of KwaZulu-Natal, Durban, KwaZulu-Natal, South Africa

## Abstract

**Background:**

Antibiotic resistance (ABR) is a growing global health concern, with inappropriate prescribing practises playing a significant role. The drug resistance index (DRI) is a composite metric used by policymakers to assess the effectiveness of antibiotic therapy, where a score of 0 indicates complete susceptibility and 100 indicating complete resistance.

**Methods:**

This study evaluated the DRI for *Escherichia coli* and *Klebsiella pneumoniae* using antibiotic consumption and resistance data from two public sector hospitals in the uMgungundlovu District in KwaZulu-Natal, between 2017 and 2019. Antibiotic consumption (ABC) data were sourced from the Provincial Department of Health for one district and one tertiary hospital, while antibiotic resistance data were obtained from a surveillance study of urinary tract and bloodstream infections. ABC was calculated using the WHO GLASS methodology, incorporating Defined Daily Dose (DDD) and bed-occupancy.

**Results:**

The DRI ranged from 55-58 for *E. coli* and and 68-88 for *K. pneumoniae*, across the study period, indicating reduced effectiveness of current empiric treatments .

**Conclusion:**

These findings highlight the urgent need to revise standard treatment guidelines at the hospital level. Strengthening national surveillance of ABR and ABC is essential to inform evidence-based policy and optimize antimicrobial stewardship.

**Disclosures:**

Sabiha Essack, B. Pharm, M. Pharm, PhD, CARB-X: Board Member|GSK: Honoraria|Reckitt (Pty.) Ltd.: Honoraria

